# Fluid accumulation and major adverse kidney events in sepsis: a multicenter observational study

**DOI:** 10.1186/s13613-022-01040-6

**Published:** 2022-07-04

**Authors:** Alessandro Mele, Emanuele Cerminara, Henrike Häbel, Borja Rodriguez-Galvez, Anders Oldner, David Nelson, Johannes Gårdh, Ragnar Thobaben, Sandra Jonmarker, Maria Cronhjort, Jacob Hollenberg, Johan Mårtensson

**Affiliations:** 1 Department of Physiology and Pharmacology, Section of Anaesthesia and Intensive Care, Karolinska Institutet, Stockholm, Sweden; 2 Instituto Di Anestesiologia E Rianimazione, Università Cattolica del Sacro Cuore, Rome, Italy; 3 Institute of Environmental Medicine, Division of Biostatistics, Karolinska Institutet, Stockholm, Sweden; 4 School of Electrical Engineering and Computer Science, KTH Royal Institute of Technology, Stockholm, Sweden; 5 Department of Perioperative Medicine and Intensive Care, Karolinska University Hospital, 171 76 Stockholm, Sweden; 6 Department of Clinical Science and Education Södersjukhuset, Section of Anaesthesia and Intensive Care, Karolinska Institute, Stockholm, Sweden; 7 Department of Anaesthesia and Intensive Care, Södersjukhuset, Stockholm, Sweden; 8 Department of Clinical Science and Education Södersjukhuset, Center for Resuscitation Science, Karolinska Institutet, Stockholm, Sweden

**Keywords:** Fluid balance, Major adverse kidney events, Intensive care, Renal replacement therapy, Sepsis, Acute kidney injury

## Abstract

**Background:**

Whether early fluid accumulation is a risk factor for adverse renal outcomes in septic intensive care unit (ICU) patients remains uncertain. We assessed the association between cumulative fluid balance and major adverse kidney events within 30 days (MAKE30), a composite of death, dialysis, or sustained renal dysfunction, in such patients.

**Methods:**

We performed a multicenter, retrospective observational study in 1834 septic patients admitted to five ICUs in three hospitals in Stockholm, Sweden. We used logistic regression analysis to assess the association between cumulative fluid balance during the first two days in ICU and subsequent risk of MAKE30, adjusted for demographic factors, comorbidities, baseline creatinine, illness severity variables, haemodynamic characteristics, chloride exposure and nephrotoxic drug exposure. We assessed the strength of significant exposure variables using a relative importance analysis.

**Results:**

Overall, 519 (28.3%) patients developed MAKE30. Median (IQR) cumulative fluid balance was 5.3 (2.8–8.1) l in the MAKE30 group and 4.1 (1.9–6.8) l in the no MAKE30 group, with non-resuscitation fluids contributing to approximately half of total fluid input in each group. The adjusted odds ratio for MAKE30 was 1.05 (95% CI 1.02–1.09) per litre cumulative fluid balance. On relative importance analysis, the strongest factors regarding MAKE30 were, in decreasing order, baseline creatinine, cumulative fluid balance, and age. In the secondary outcome analysis, the adjusted odds ratio for dialysis or sustained renal dysfunction was 1.06 (95% CI 1.01–1.11) per litre cumulative fluid balance. On separate sensitivity analyses, lower urine output and early acute kidney injury, respectively, were independently associated with MAKE30, whereas higher fluid input was not.

**Conclusions:**

In ICU patients with sepsis, a higher cumulative fluid balance after 2 days in ICU was associated with subsequent development of major adverse kidney events within 30 days, including death, renal replacement requirement, or persistent renal dysfunction.

**Supplementary Information:**

The online version contains supplementary material available at 10.1186/s13613-022-01040-6.

## Background

Fluid resuscitation is cornerstone treatment of haemodynamic instability during the early phase of sepsis [1]. Conventional fluid resuscitation combined with the administration of maintenance fluids, drug diluents, and nutrition [2, 3] often leads to a degree of fluid accumulation, typically reaching 2–4 l on average after 2 days in the intensive care unit (ICU) [4–7].

Experimental data propose a possible link between fluid accumulation and renal parenchymal oedema, which may induce congestion and impaired glomerular filtration rate [8–10]. This is supported by data from observational studies and one randomized pilot trial indicating that greater fluid accumulation is associated with worsening kidney function and/or mortality [11–14].

In addition to fluid volume accumulation, fluid composition and adjunct ICU therapies may contribute to adverse renal outcomes. For example, data from large randomized trials suggest that fluid resuscitation with hydroxyethyl starch [5, 6] or chloride-rich solutions [15, 16] significantly increases the risk of adverse renal outcomes. Management of vasopressor therapy to achieve a target mean arterial pressure (MAP) may also modify the risk in subgroups of patients [17, 18]. Finally, the use of nephrotoxic antibiotics, commonly used in patients with severe sepsis, add further insult to the kidneys.

Since, greater illness severity is associated with the administration of larger fluid volumes, more intense cardiovascular support, and greater use of potentially nephrotoxic drugs, the relative importance of fluid accumulation with respect to adverse renal outcomes is an important clinical question that remains uncertain. Accordingly, we conducted an observational multi-centre study to assess the independent association between early fluid accumulation and major adverse kidney events, a composite of death, dialysis, or sustained renal dysfunction, within 30 days (MAKE30) in ICU patients with sepsis. In addition, we aimed to assess the relative importance of fluid accumulation and other potential factors for MAKE30 development. We hypothesized that the degree of fluid accumulation after 2 days in ICU would be associated with subsequent MAKE30 after controlling for illness severity, level of cardiovascular support, and nephrotoxic drug use in such patients.

## Methods

The study was approved by the Stockholm Ethical Review Board (approval number 2018/858-31) with a waiver of informed consent.

### Study design and participants

We conducted a multicenter, retrospective cohort study of patients admitted to five ICUs in three hospitals in the Stockholm Region between June 2005 and August 2018. We included adult (16 years or older) patients admitted with a diagnosis of sepsis (International Classification of Diseases, 10th revision [ICD-10] code A41.9) severe sepsis (ICD-10 code R65.1) or septic shock (ICD-10 code R57.2) on their first ICU admission. We excluded patients with end-stage renal disease and patients who died, who were discharged or who received renal replacement therapy within the first two days in ICU. We also excluded patients without data on fluid balance or creatinine.

### Data collection

We collected data from the electronic ICU patient data management system Centricity Critical Care (GE Healthcare, Chicago, IL) and the hospital electronic health record system Take Care (CompuGroup Medical, Koblenz, Germany). From the Centricity Critical Care data warehouse, we included demographic data, admission diagnoses and comorbidity data (ICD-10 codes), information on administered intravenous fluids, enteral and parenteral nutrition, blood products and drugs (including continuous drug infusions), total daily fluid input and output, arterial blood gas data, laboratory data, timing of invasive mechanical ventilation and renal replacement therapy initiation, and invasive mean arterial pressure (MAP) data (recorded every second minute on average). From the Take Care data warehouse, we included information on pre-admission and post-discharge creatinine and death date.

### Exposure variables

Primary exposure was the cumulative fluid balance (cumulative total fluid input minus cumulative total fluid output) during the first two days in ICU (exposure period). We also included baseline exposure variables (demographics, baseline creatinine, comorbidities, location before ICU admission, admission hospital, and admission year) and the following variables collected during the exposure period: variables reflecting illness severity (vasoactive support, MAP duration below 65 mmHg, mechanical ventilation, lactate, bilirubin, and platelet count), exposure to potentially nephrotoxic drugs (vancomycin, aminoglycosides, and hydroxyethyl starch), level of systemic inflammation (C-reactive protein level), and other variables potentially associated with both the primary exposure and outcome (cumulative chloride dose, highest chloride level, furosemide administration, and red blood cell transfusion). Definition and categorization of exposure variables are presented in Additional file 1. Filtering and processing of MAP data is described in Additional file 1. Early (first 48 h) acute kidney injury (AKI) was defined based on changes in plasma creatinine and/or oliguria according to the Kidney Disease: Improving Global Outcomes (KDIGO) criteria [19].

### Outcomes

The primary outcome was major adverse kidney events occurring after the exposure period until 30 days after ICU admission (MAKE30). MAKE30 was defined as the composite of death, initiation of renal replacement therapy, or sustained renal dysfunction (a last inpatient plasma creatinine level [within 30 days] ≥ 200% of the baseline level) [15, 20]. We used the first creatinine level obtained during the exposure period as baseline. When creatinine was missing during this period, we selected the most recent creatinine level obtained within one year before ICU admission. Finally, if baseline creatinine was missing with these two approaches, it was estimated using the following equations [21]:

$$ {\text{Creatinine}}\left( {\mu {\text{mol}}/{\text{l}}} \right) =\, 88.4 \times \left( {0.74 - 0.2 + 0.003 \times {\text{age}}} \right)\,{\text{in}}\,{\text{females}},\,{\text{and}} $$

$$ {\text{Creatinine}}\,\left( {\upmu {\text{mol/l}}} \right) = 88.4 \times \left( {0.74 + 0.003 \times {\text{age}}} \right)\,{\text{in}}\,{\text{males}}{.} $$

The secondary outcome was the composite of renal replacement therapy initiation or sustained renal dysfunction.

### Statistical analysis

The association between the log-odds for suffering MAKE30 and the cumulative fluid balance during the exposure period was estimated using univariable and multivariable logistic regression analysis. The robust sandwich estimator was used to estimate standard errors and two-sided *p*-values were reported. We considered all exposure variables presented in Additional file 1 for inclusion in the multivariable model. To select variables for the multivariable regression model, hierarchical backwards selection was conducted using the Wald test and a significance level of 0.2. Model performance was evaluated by estimating the area under the receiver operating characteristic (ROC) curve and by visual inspection of the residuals. In a sensitivity analysis, we reassessed the association between cumulative fluid balance and MAKE30 after replacing baseline creatinine with early AKI in the model. To better understand the contribution of fluid input and urine output, respectively, to the association with MAKE30, we performed an additional sensitivity analysis including these variables instead of the cumulative fluid balance. We estimated the relative importance of exposure variables using binary splits calculated by recursive partitioning implemented in the ‘rpart’ R package [22]. An overall measure of variable importance is the sum of the goodness of split measures plus adjusted agreement for all splits (details in Additional file 1). In a sensitivity analysis, restricted cubic splines of the cumulative fluid balance were used in a second multivariable model to confirm that a linear relationship between the outcome and the cumulative fluid balance can be assumed. Knot locations were based on Harrell's recommended percentiles. In a further sensitivity analysis on variable selection, manual forward selection was performed by adding groups of variables one at a time. The different forward selection models were compared by their Akaike information criterion (AIC). In the secondary outcome analyses we included both survivors and non-survivors at 30 days; patients not receiving renal replacement therapy and not having a last plasma creatinine level ≥ 200% of the baseline level before death were considered event free. Those who received renal replacement therapy or had a last plasma creatinine level ≥ 200% of the baseline level before death were considered to meet the event. All analyses were conducted using STATA version 16.1 for Windows (StataCorp LLC, College Station, Texas 77,845 USA) unless otherwise stated. Data were analysed as complete cases. A two-sided *p* value less than 0.05 was considered statistically significant.

## Results

### Patients

**Fig. 1:**
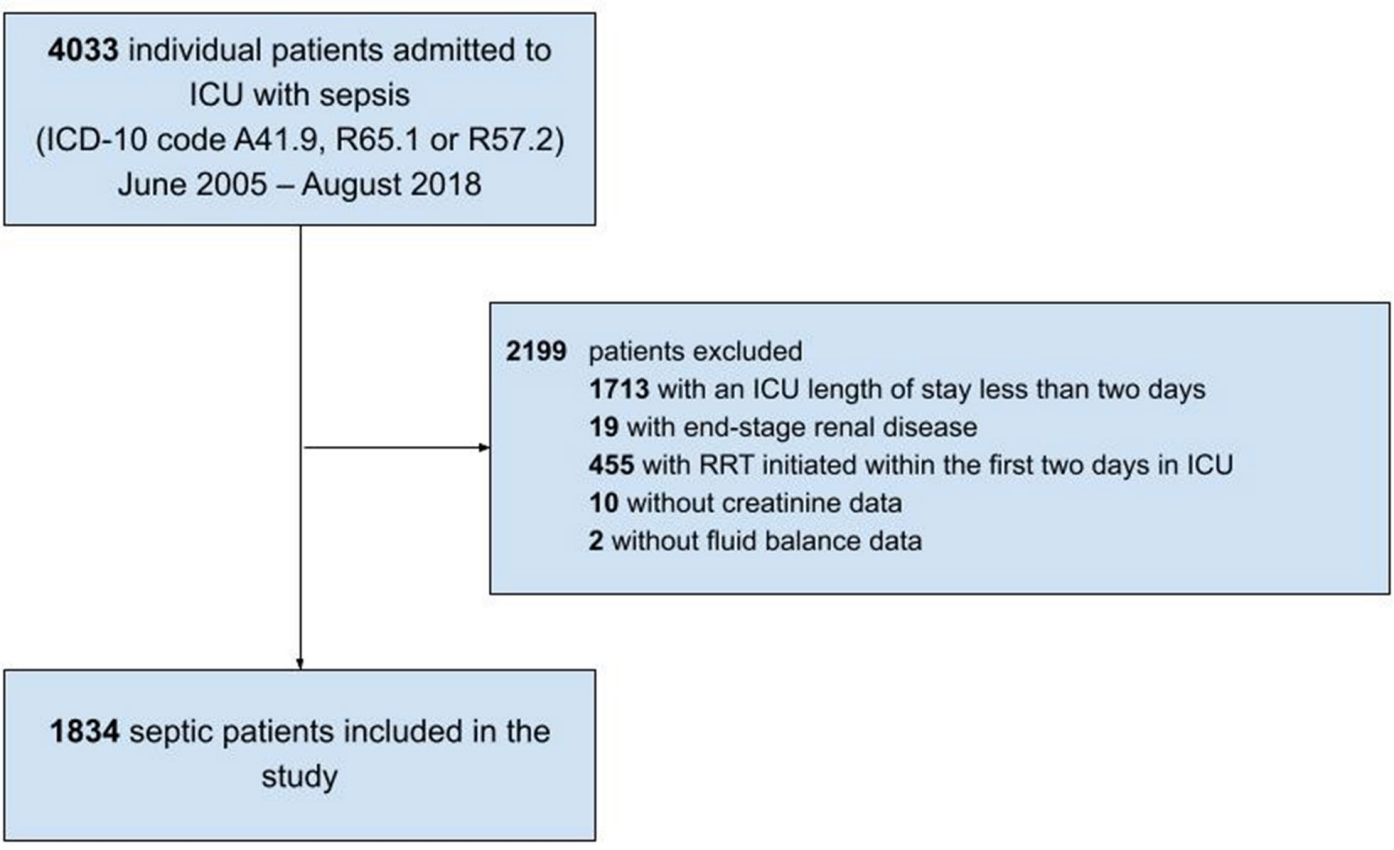
Patient selection. *ICU* intensive care unit, *ICD-10* International Classification of Diseases, 10th revision; *RRT* renal replacement therapy

A total of 4033 patients were admitted to ICU with a diagnosis of sepsis (according to the ICD-10 coding system) between June 2005 and August 2018. We excluded 2199 patients who were discharged or received renal replacement therapy within the first 2 days in ICU, had end-stage renal disease, or had missing creatinine or fluid balance data. Therefore, we included 1834 patients (Fig. 1); 55.7% males; median (IQR) age 67 (56–75) years. Overall, 519 (28.3%) patients developed MAKE30; 417 (22.7%) died within 30 days; 128 (7.0%) received renal replacement therapy [85 (6.0%) among survivors and 43 (10.3%) among non-survivors; 38 (2.1%] had a last inpatient plasma creatinine level ≥ 200% of the baseline level without renal replacement therapy [7 (1.2%) among survivors and 21 (5.0%) among non-survivors](Table 1). The last inpatient plasma creatinine was available after a median (IQR) of 20 (10–30) days after ICU arrival in MAKE30 survivors.

**Table 1:** Outcomes

Outcome	All patients (*n* = 1834)
Primary outcome	
Major adverse kidney events within 30 days	519/1834 (28.3%)
Components of primary outcome	
Death within 30 days	417/1834 (22.7%)
Renal replacement therapy	128/1834 (7.0%)
Among survivors	85/1417 (6.0%)
Among non-survivors	43/417 (10.3%)
Last inpatient plasma creatinine level ≥ 200% of baseline without renal replacement therapy	38/1834 (2.1%)
Among survivors	17/1417 (1.2%)
Among non-survivors	21/417 (5.0%)

Values are *n* (%)

As compared to patients without MAKE30, patients with MAKE30 were older, weighed less, had higher baseline creatinine, and were more likely to have chronic liver disease, chronic cardiac disease, and/or immune deficiency. We observed small but statistically significant differences in location before ICU admission, and admission hospital between groups (Table 2).

**Table 2:** Baseline characteristics

Characteristic	All (*n* = 1834)	No MAKE30 (*n* = 1315)	MAKE30 (*n* = 519)	*p* value
Age, years	67 (56–75)	66 (55–74)	70 (60–77)	< 0.001
Male sex	1021 (55.7%)	740 (56.3%)	281 (54.1%)	0.41
Body weight on ICU admission, kg^a^	78 (66–90)	78 (67–91)	75 (64–88)	0.005
Baseline creatinine, µmol/l	123 (81–189)	119 (80–181)	132 (84–214)	0.001
Baseline creatinine approach				0.51
Obtained from first creatinine level in ICU	1814 (98.9%)	1303 (99.1%)	511 (98.5%)	
Obtained from pre-admission creatinine level	5 (0.3%)	3 (0.2%)	2 (0.4%)	
Obtained from estimated creatinine level	15 (0.8%)	9 (0.7%)	6 (1.2%)	
Chronic liver disease	152 (8.3%)	84 (6.4%)	68 (13.1%)	< 0.001
Chronic cardiac disease	307 (16.7%)	203 (15.4%)	104 (20.0%)	0.017
Chronic respiratory disease	205 (11.2%)	138 (10.5%)	67 (12.9%)	0.14
Immune deficiency	596 (32.5%)	382 (29.0%)	214 (41.2%)	< 0.001
Location before ICU admission				< 0.001
Other ICU	83 (4.5%)	67 (5.1%)	16 (3.1%)	
Emergency department	511 (27.9%)	370 (28.1%)	141 (27.2%)	
Recovery	85 (4.6%)	77 (5.9%)	8 (1.5%)	
Operating room	202 (11.0%)	151 (11.5%)	51 (9.8%)	
High dependency unit	82 (4.5%)	50 (3.8%)	32 (6.2%)32 (6.2%)	
Ward	871 (47.5%)	600 (45.6%)	271 (52.2%)	
Hospital				0.039
A	552 (30.1%)	409 (31.1%)	143 (27.6%)	
B	511 (27.9%)	345 (26.2%)	166 (32.0%)	
C	770 (42.0%)	561 (42.7%)	209 (40.3%)	

Data are *n* (%) or median (IQR)

*MAKE30* major adverse kidney events within 30 days; *ICU* intensive care unit

^a^Data available for 1289 (98%) patients in the No MAKE30 group and 509 (98%) patients in the MAKE30 group

### Process of care

Process of care and biochemical characteristics during the exposure period are shown in Table 3. During the exposure period, median (IQR) cumulative fluid balance was 5258 (2777–8132) ml in the MAKE30 group and 4127 (1864–6755) ml in the no MAKE30 group (*p* < 0.001). Total urine output was lower in the MAKE30 group whereas total fluid input was similar in the two groups (Table 4). Cumulative fluid balance by admission year is displayed in Additional file 2: Figure S1. Commonly used resuscitation fluids (Ringer’s solutions, albumin solutions, hydroxyethyl starch solutions, dextran solutions and gelatin solutions) contributed to approximately 50% of total fluid intake in both groups during the exposure period (Table 4). Early AKI was more common in the MAKE30 group (44.3%) than in the no MAKE30 group (25.7%, *p* < 0.001). Patients with MAKE30 were more likely to receive a noradrenaline infusion rate above 0.20 µg/kg/min (*p* < 0.001) and to receive inotropic support (*p* = 0.005), vancomycin (*p* = 0.03), furosemide (*p* = 0.006), and/or invasive mechanical ventilation (*p* = 0.002). In addition, highest blood lactate and bilirubin levels were higher, whereas lowest platelet count was lower in the MAKE30 group (Table 3).

**Table 3:** Process of care and biochemical characteristics during exposure period

Characteristic	All (*n* = 1834)	No MAKE30 (*n* = 1315)	MAKE30 (*n* = 519)	*p* value
Cumulative fluid balance, ml	4503 (2140–7158)	4127 (1864–6755)	5258 (2777–8132)	< 0.001
Acute kidney injury^a^	568 (31.0%)	338 (25.7%)	230 (44.3%)	< 0.001
Urine output criteria^b^	224 (12.3%)	133 (10.2%)	91 (17.8%)	< 0.001
Creatinine criteria^c^	443 (24.2%)	253 (19.2%)	190 (36.6%)	< 0.001
Cumulative chloride dose				0.86
< 500 mmol	370/1834 (20.2%)	264/1315 (20.1%)	106/519 (20.4%)	
500–999 mmol	884/1834 (48.2%)	639/1315 (48.6%)	245/519 (47.2%)	
≥ 1000 mmol	580/1834 (31.6%)	412/1315 (31.3%)	168/519 (32.4%)	
Highest noradrenaline infusion rate				< 0.001
≤ 0.10 µg/kg/min	533/1834 (29.1%)	405/1315 (30.8%)	128/519 (24.7%)	
0.11–0.20 µg/kg/min	519/1834 (28.3%)	389/1315 (29.6%)	130/519 (25.0%)	
> 0.20 µg/kg/min	782/1834 (42.6%)	521/1315 (39.6%)	261/519 (50.3%)	
Vasopressin infusion	61/1834 (3.3%)	39/1315 (3.0%)	22/519 (4.2%)	0.17
Inotropic support^d^	388/1834 (21.2%)	256/1315 (19.5%)	132/519 (25.4%)	0.005
Duration of MAP < 65 mmHg				0.17
< 5 h	753/1805 (41.7%)	544/1293 (42.1%)	209/512 (40.8%)	
5–10 h	413/1805 (22.9%)	307/1293 (23.7%)	106/512 (20.7%)	
> 10 h	639/1805 (35.4%)	442/1293 (34.2%)	197/512 (38.5%)	
Duration of MAP < 55 mmHg				< 0.001
< 5 h	1680 (93.1%)	1221 (94.4%)	459 (89.7%)	
5–10 h	75 (4.2%)	47 (3.6%)	28 (5.5%)	
> 10 h	50 (2.8%)	25 (1.9%)	25 (4.9%)	
Vancomycin administration	134/1834 (7.3%)	85/1315 (6.5%)	49/519 (9.4%)	0.027
Aminoglycoside administration	341/1834 (18.6%)	244/1315 (18.6%)	97/519 (18.7%)	0.95
Hydroxyethyl starch administration	289/1834 (15.8%)	212/1315 (16.1%)	77/519 (14.8%)	0.74
Furosemide administration	1292/1834 (70.4%)	902/1315 (68.6%)	390/519 (75.1%)	0.006
Red blood cell transfusion	655/1834 (35.7%)	458/1315 (34.8%)	197/519 (38.0%)	0.16
Invasive mechanical ventilation during exposure period	982/1834 (53.5%)	675/1315 (51.3%)	307/519 (59.2%)	0.002
Blood lactate > 2 mmol/l	1419/1821 (77.9%)	994/1305 (76.2%)	425/516 (82.4%)	0.004
Highest chloride level				0.32
< 108 mmol/l	457/1821 (25.1%)	316/1305 (24.2%)	141/516 (27.3%)	
108–111 mmol/l	622/1821 (34.2%)	446/1305 (34.2%)	176/516 (34.1%)	
> 111 mmol/l	742/1821 (40.7%)	543/1305 (41.6%)	199/516 (38.6%)	
Highest C-reactive protein, mg/l	298 (213–362)	300 (222–362)	291 (191–358)	0.11
Highest bilirubin level				< 0.001
< 20 µmol/l	897/1722 (52.1%)	678/1232 (55.0%)	219/490 (44.7%)	
20–32 µmol/l	388/1722 (22.5%)	277/1232 (22.5%)	111/490 (22.7%)	
> 32 µmol/l	437/1722 (25.4%)	277/1232 (22.5%)	160/490 (32.7%)	
Lowest platelet count				< 0.001
≥ 150	770/1804 (42.7%)	609/1294 (47.1%)	161/510 (31.6%)	
100–149	383/1804 (21.2%)	274/1294 (21.2%)	109/510 (21.4%)	
< 100	651/1804 (36.1%)	411/1294 (31.8%)	240/510 (47.1%)	

Values are n/n with available data (%) or median (IQR)

*MAKE30* major adverse kidney events within 30 days, *MAP* mean arterial pressure

^a^Defined based on changes in plasma creatinine and/or oliguria according to the Kidney Disease: Improving Global Outcomes (KDIGO) criteria

^b^Urine output < 0.5 ml/kg/hour for at least 6 consecutive hours

^c^Increase in plasma creatinine to ≥ 1.5 times baseline or by 26.5 µmol/l or to ≥ 353.6 µmol/l

^d^Inotropic support was defined as a continuous infusion of either adrenaline, dobutamine, levosimendan or milrinone

**Table 4:** Type and volume of administered fluids and urine output during the first 2 days in ICU

Fluid type	No MAKE30 (*n* = 1315)			MAKE30 (*n* = 519)		
	No. of patients^a^	Median (IQR) volume (ml)^b^	% of total fluid volume^c^	No. of patients^a^	Median (IQR) volume (ml)^b^	% of total fluid volume^c^
Total fluid input	1315	8395 (6233–11,130)	100	519	8559 (6245–11,531)	100
Resuscitation fluids						
Ringer’s solution	1235	3500 (2000–5357)	41.7	481	3460 (2000–5670)	41.0
Albumin 40 or 50 g/l	359	500 (481–1000)	2.6	139	750 (490–1289)	2.8
Albumin 200 g/l	498	200 (100 – 348)	1.2	240	200 (100–400)	1.5
Hydroxyethyl starch	212	963 (500–1014)	1.8	77	1000 (500–1461)	1.7
Dextran	111	600 (500–1000)	0.8	28	733 (500–1000)	0.6
Gelofusine	117	1500 (500–2000)	1.4	30	1000 (500–1500)	0.8
	Proportion of total fluid input from resuscitation fluids		49.5	Proportion of total fluid input from resuscitation fluids		48.4
Non resuscitation fluids						
0.9% saline	1099	432 (152–921)	6.9	451	506 (201–1178)	9.2
Glucose 25–200 g/l	1283	2106 (1428–2891)	24.3	509	2082 (1316–2954)	23.6
Glucose 300–500 g/l	6	25 (20–40)	< 0.1	9	30 (20–90)	< 0.1
Red blood cells	458	500 (250–750)	2.6	197	500 (250–750)	2.8
Plasma	342	1000 (500–1750)	4.3	147	750 (500–1500)	3.9
Platelets	123	600 (300–650)	0.6	100	600 (300–900)	1.5
Parenteral nutrition	909	182 (12–1096)	5.2	353	112 (11.8–1239)	5.3
Enteral (oral or nasogastric) fluids	435	710 (325–1300)	3.5	125	425 (200–800)	1.9
Enteral nutrition (IQR)	345	326 (163–566)	1.4	136	295 (171–495)	1.4
Electrolyte solutions	1215	78 (36–143)	1.2	474	94 (39–199)	1.6
Other fluids	109	100 (40–280)	0.3	64	169 (50–462)	0.4
	Proportion of total fluid input from non-resuscitation fluids		50.5	Proportion of total fluid input from non-resuscitation fluids		51.6
Total urine output	1305	4215 (2821–6098)		511	3081 (1580–4787)	

^a^Total number of patients who received the fluid type presented on each row

^b^Median (IQR) delivered volume of each fluid (among those who received the specific fluid type)

^c^Proportion of total fluid intake in the No MAKE30 group and MAKE30 group, respectively

### Primary outcome analyses

**Fig. 2:**
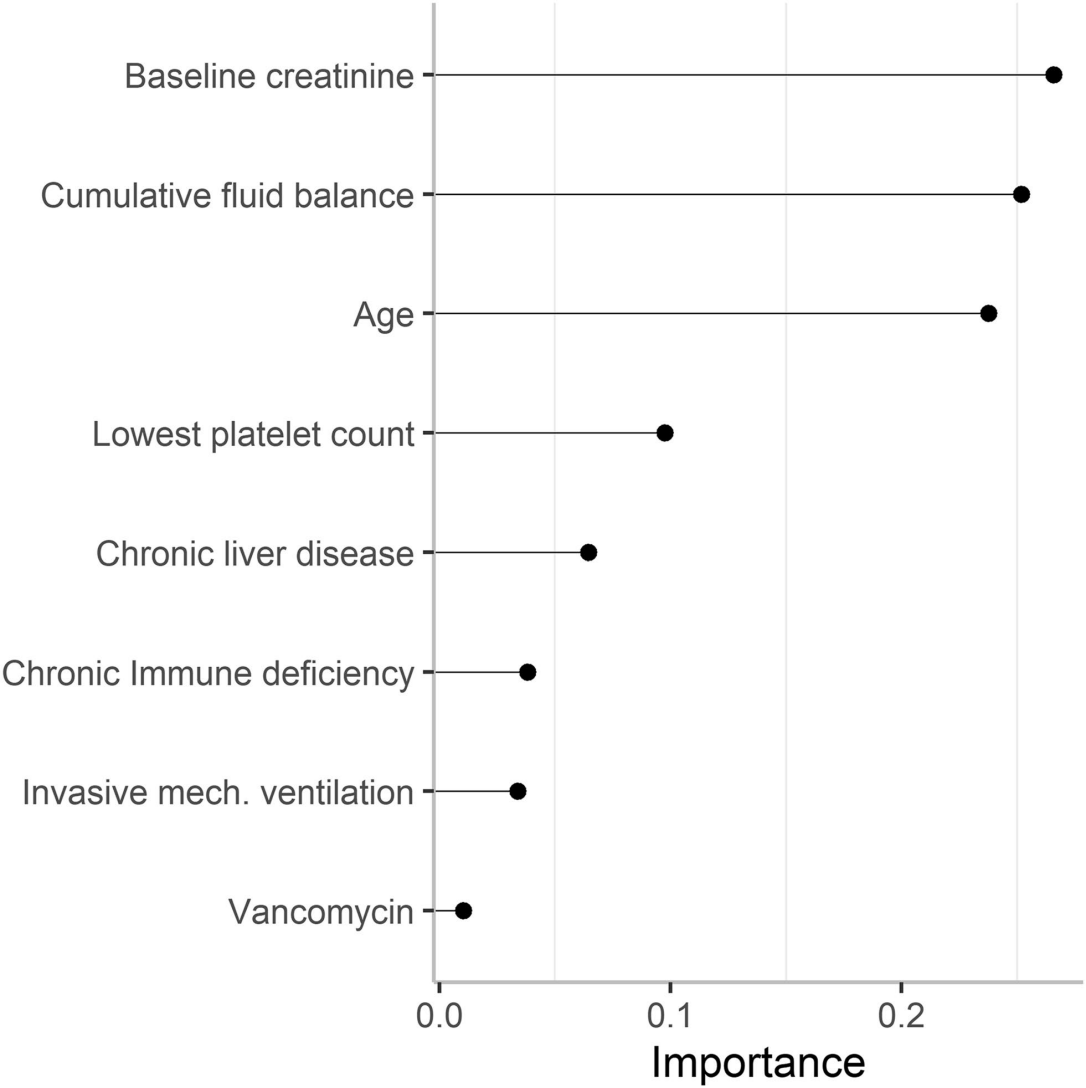
Relative importance of factors associated with major adverse kidney events within 30 days

Table 5 shows the adjusted odds ratio (OR) for MAKE30 using hierarchical backwards selection of variables among the 1641 patients with complete data on covariates. The adjusted OR per one litre increase in cumulative fluid balance was 1.05 (95% CI 1.02–1.09). In addition, higher age, higher baseline creatinine, chronic liver disease, immune deficiency, vancomycin therapy, lower platelet count, and invasive mechanical ventilation were independently associated with increased risk of MAKE30. The adjusted OR for cumulative fluid balance was 1.04 (95% CI 1.003–1.07) when adjusting for early AKI instead of baseline creatinine (Additional file 2: Table S1). The adjusted ORs per one litre increase in total fluid input and total urine output were 0.97 (95% CI 0.94–1.01) and 0.84 (95% CI 0.79–0.89), respectively (Additional file 2: Table S2). Forward selection logistic regression analysis did not substantially alter the association between cumulative fluid balance and MAKE30 (Additional file 2: Table S3). On univariable and multivariable cubic spline analyses, we observed a linear increase in the OR for MAKE30 with increasing cumulative fluid balance (Additional file 2: Figure S2). Figure 2 displays the exposure variables with greatest importance with regard to MAKE30. The three strongest variables were baseline creatinine, cumulative fluid balance, and age.

**Table 5:** Multivariable logistic regression analysis showing the association with major adverse kidney events within 30 days

Variable	Unadjusted odds ratio (95% CI)	*p *value	Adjusted odds ratio^a^ (95% CI)	*p* value
Cumulative fluid balance, litre	1.08 (1.05–1.11)	< 0.001	1.05 (1.02–1.09)	0.003
Age, year	1.02 (1.01–1.03)	< 0.001	1.03 (1.02–1.04)	< 0.001
Male sex	0.92 (0.75–1.13)	0.41	0.86 (0.67–1.11)	0.25
Body weight, kg	0.99 (0.99–1.00)	0.01	0.99 (0.99–1.00)	0.09
Baseline creatinine, per 10 µmol/l	1.00 (1.00–1.01)	0.02	1.01 (1.00–1.02)	0.03
Chronic liver disease	2.21 (1.58–3.10)	< 0.001	1.86 (1.23–2.82)	0.003
Chronic cardiac disease	1.37 (1.06–1.78)	0.02	1.32 (0.96–1.82)	0.09
Chronic respiratory disease	1.26 (0.93–1.73)	0.14	1.19 (0.82–1.73)	0.35
Immune deficiency	1.71 (1.39–2.12)	< 0.001	1.62 (1.25–2.10)	< 0.001
Duration with MAP < 65 mmHg				
< 5 h	1.00		1.00	
5–10 h	0.90 (0.68–1.18)	0.44	0.74 (0.54–1.01)	0.06
> 10 h	1.16 (0.92–1.46)	0.21	0.86 (0.66–1.13)	0.29
Vancomycin therapy	1.51 (1.04–2.18)	0.03	1.75 (1.12–2.74)	0.01
Aminoglycoside therapy	1.00 (0.78–1.31)	0.95	0.88 (0.65–1.20)	0.42
Hydroxyethyl starch administration	0.95 (0.72–1.26)	0.74	0.96 (0.66–1.38)	0.81
Highest C-reactive protein, mg/l	1.00 (1.00–1.00)	0.23	1.00 (1.00–1.00)	0.44
Highest chloride level				
< 108 mmol/l	1.00		1.00	
108–111 mmol/l	0.88 (0.68–1.15)	0.36	0.90 (0.66–1.23)	0.51
> 111 mmol/l	0.82 (0.64–1.06)	0.13	0.76 (0.56–1.04)	0.08
Highest lactate level				
< 2 mmol/l	1.00		1.00	
≥ 2 mmol/l	1.46 (1.13–1.89)	0.004	1.05 (0.76–1.45)	0.77
Highest bilirubin level				
< 20 µmol/l	1.00		1.00	
20–32 µmol/l	1.24 (0.95–1.62)	0.11	1.03 (0.76–1.39)	0.84
> 32 µmol/l	1.79 (1.40–2.29)	< 0.001	1.31 (0.97–1.75)	0.08
Lowest platelet count				
≥ 150	1.00		1.00	
100–149	1.50 (1.14–1.99)	0.004	1.60 (1.16–2.21)	0.004
< 100	2.21 (1.75–2.80)	< 0.001	2.05 (1.53–2.74)	< 0.001
Invasive mechanical ventilation	1.37 (1.12–1.69)	0.003	1.69 (1.31–2.19)	< 0.001

^a^The model included 1641 patients with complete data and was also adjusted for admission source, admission hospital, and admission year. Model area under the receiver operating characteristics curve 0.72

### Secondary outcome analyses

A total of 166 (9.1%) patients met the secondary outcome event (102 survivors and 64 non-survivors) and 1668 (90.9%) were event free (1315 survivors and 353 non-survivors) (Table 1). The adjusted OR for the secondary outcome per one litre increase in cumulative fluid balance was 1.06 (95% CI 1.01–1.11) using hierarchical backwards selection of variables (Additional file 2: Table S4). Forward selection logistic regression analysis did not substantially alter the association between cumulative fluid balance and the secondary outcome (Additional file 2: Table S5).

## Discussion

### Key findings

Whether early fluid accumulation is a risk factor for adverse renal outcomes in septic patients requiring ICU treatment is an important clinical question that remains uncertain. The main finding of this observational multicenter study is that cumulative fluid balance was significantly associated with major adverse kidney events within 30 days (MAKE30), defined as a composite of death, dialysis, or sustained renal dysfunction. This finding was independent of baseline patient characteristics, degree of illness severity, cardiovascular support, duration of MAP below 65 mmHg, chloride exposure, nephrotoxic drug exposure, and invasive ventilatory support. On relative importance analysis of independent variables, the strongest factors regarding MAKE30 were baseline creatinine, cumulative fluid balance, and age. The second main finding of the study is that the administration of non-resuscitation fluids significantly contributes to the early fluid input and cumulative fluid balance in septic ICU patients. The third main finding of the study is that the urine output component of the cumulative fluid balance rather than the fluid input component is responsible for the association with MAKE30.

### Relationship with previous studies

Our findings are to some extent, but not entirely, in line with previous studies. Observational data suggest a positive relationship between greater fluid accumulation in the ICU and death among sepsis patients [23–25], but such findings are inconsistent [26]. Contemporary studies assessing the effect of fluid accumulation in sepsis with regard to renal outcomes are scarce and have also shown conflicting evidence. Two observational studies (including 113 and 107 patients, respectively) found no independent association between the 24-h fluid balance and risk of worsening AKI [27, 28]. Conversely, in the Conservative vs. Liberal Approach to fluid therapy of Septic Shock in Intensive Care (CLASSIC) pilot trial, the use of a restrictive resuscitation fluid protocol attenuated AKI progression. However, their cumulative fluid balance did not differ from those exposed to conventional fluid resuscitation, suggesting that non-resuscitation fluid contributed significantly to overall fluid accumulation [14].

This lack of robust evidence underpins the debate on whether fluid accumulation per se contributes to adverse outcomes or is simply a marker of the degree of illness severity. Assessing the effect of fluid accumulation on renal outcomes is particularly challenging as AKI in itself is a major contributor to fluid gain (risk of reverse causation). The potential problem of death as competing risk for adverse renal events should also be acknowledged. In the present study, we set out to minimize these issues by (a) assessing an exposure period (first 2 days in ICU) to allow sufficient fluid accumulation before the outcome was allowed to occur; (b) considering potentially important patient-related, treatment-related, and illness-severity-related confounders in the analysis; (c) including death in the composite outcome (MAKE30) as recommended by consensus statements [29]. Our analysis showed that not only was the association between early fluid accumulation and MAKE30 independent of several relevant confounders, but also a relatively strong factor when compared with other potential factors associated with MAKE30. We acknowledge that mortality was the dominant component of MAKE30. However, our secondary outcome analyses suggest that fluid accumulation is also associated with “pure” renal outcomes.

During the exposure period, approximately half of the total fluid input came from non-resuscitation fluids, a finding consistent with observations from other cohorts. For example, in a recent study including 1229 sepsis patients from one ICU in Belgium, non-resuscitation fluid administration was responsible for more than 50% of the cumulative fluid balance after 48 ICU hours [2]. In another single-centre study from the US, medication diluents contributed to more than 60% of the total intravenous fluid input during the ICU course [3]. Finally, in a Swedish-Canadian survey of 200 patients with septic shock, the median volume of resuscitation and non-resuscitation fluids was similar on day one in ICU [30].

Importantly, the higher cumulative fluid balance in our MAKE30 group was a consequence of lower urine output rather than greater fluid input. Moreover, in our adjusted sensitivity analysis, lower urine output was independently associated with higher odds of MAKE30, whereas the OR for fluid input remained insignificant. Low urine output is a physiological consequence of pending AKI, which in itself is linked to MAKE30 (see Additional file 2: Table S1). Low urine output is also a common trigger for fluid administration [31], and contributes to fluid accumulation. Our observed relationship between urine output (and cumulative fluid balance) and MAKE30 may therefore reflect a degree of reverse causation. However, although adjustment for early AKI somewhat weakened the association between fluid balance and MAKE30, we observed a statistically significant signal of harm (Additional file 2: Table S1). This raises the question of whether a more intense fluid removal strategy, perhaps in combination with fluid restriction, has the potential to improve renal outcomes in ICU patients. To date, evidence to support ‘forced’ fluid removal is limited to pilot trials including AKI patients only or patients in the post-resuscitation phase. In one pilot trial including AKI patients without RRT, randomization (after 12–72 h in ICU) to combined fluid restriction and targeted fluid removal (using diuretics) resulted in lower cumulative fluid balance, and fewer patients requiring RRT but without a significant effect on AKI duration [32]. In another trial including mechanically ventilated, hemodynamically stabilized patients with an in-ICU weight gain of ≥ 3%, protocolized diuretic therapy attenuated fluid accumulation and the occurrence of worsening AKI [33].

### Study implications

Our findings imply that the administration of non-resuscitation fluids significantly contributes to the early fluid input and cumulative fluid balance in septic ICU patients. Moreover, they imply that the degree of fluid accumulation after 2 days in ICU is associated with adverse renal outcomes as reflected by an increased risk of major adverse kidney events within 30 days. Several potential mechanisms may explain this relationship. First, we demonstrate that decreased urine output is the main determinant of fluid accumulation and that both a lower urine output and early AKI are associated with MAKE30, which implies a degree of reverse causation. Second, excessive fluid administration in the setting of sepsis-induced capillary leakiness likely facilitates a degree of intrarenal oedema causing increased subcapsular pressure and, in extreme cases, renal compartment syndrome [8]. Third, fluid accumulation may result in elevated central venous pressure and, consequently, a reduction in the transrenal pressure difference (mean arterial pressure minus central venous pressure) that dictates renal blood flow [34]. Finally, experimental human data suggest that fluid-induced hemodilution is related to renal oxygen supply–demand mismatch, which may add further insult to the kidneys [35].

Our observational data do not provide evidence and guidance for optimal fluid management in sepsis and septic chock. Nonetheless, our findings support further assessment of strategies to attenuate fluid accumulation in septic patients treated in the ICU. Such strategies may include reduced administration of both resuscitation (e.g. the CLASSIC trial, ClinicalTrials.gov Identifier: NCT03668236) and/or non-resuscitation fluids (e.g. the OPTIFLUID trial, ClinicalTrials.gov Identifier: NCT04947904) and/or efforts to increase fluid removal.

### Strengths and limitations

Our study has strengths. We studied a previously unexplored association between early fluid accumulation and MAKE30 in adult ICU patients with sepsis. We used data from five ICUs, from three large university hospitals, involving almost 2000 patients, which provides a degree of external validity for applying our findings to similar settings. In addition, as to present the strengths of independent associations between exposure variables and outcomes (using adjusted odds ratios), we also provide estimates of the importance of exposure variables. Finally, our findings were consistent in sensitivity analyses lending robustness to our findings.

Our study has limitations. First, it is an observational study and can only describe associations. Second, we used ICD-diagnoses of sepsis rather than Sepsis-3 criteria. Our approach likely underestimated the true sepsis incidence. In fact, recent Swedish data indicate that only one-third of septic ICU patients received the sepsis diagnosis at discharge [36]. Third, we did not assess changes in exposure variables beyond the first 2 days in ICU. However, our approach minimizes the risk of reverse causation when interpreting the results. Fourth, we lack data on pre-admission fluid balance and were therefore unable to assess fluid status at baseline. Fifth, we lack data on fluid responsiveness and acknowledge that the calculated fluid balance may not correlate with intravascular-fluid status. Finally, the indication for the administration of Ringer’s solutions, albumin solutions and synthetic colloids (e.g. volume resuscitation, maintenance, albumin replacement) was unknown. Therefore, the proportions of total fluid input from resuscitation fluids should be interpreted with caution. These fluids are, however, typically used for volume expansion at the included ICUs.

## Conclusions

In adult patients with sepsis admitted to five ICUs in Sweden, a higher cumulative fluid balance after two days in ICU was independently associated with major adverse kidney events within 30 days, including death, renal replacement requirement, or persistent renal dysfunction.

## Supplementary Information

**Additional file 1:** Detailed list of exposure variables; Filtering and processing of mean arterial pressure data; Description of Variable Importance analysis.

**Additional file 2:** **Figure S1.** Cumulative fluid balance by admission year, **Figure S2.** Restricted cubic spline curves, **Table S1.** Backwards selection logistic regression adjusted for early AKI, **Table S2.** Backwards selection logistic regression adjusted for fluid input and urine output, **Table S3.** Forward selection logistic regression (association with MAKE30), **Table S4.** Backwards selection logistic regression (association with RRT or sustained renal dysfunction), **Table S5.** Forward selection logistic regression (association with RRT or sustained renal dysfunction).

## Data Availability

The datasets used and/or analysed during the current study are available from the corresponding author on reasonable request.

## Abbreviations

AIC: Akaike information criterion
CLASSIC: Conservative vs. Liberal Approach to fluid therapy of Septic Shock in Intensive Care
ICD-10: International Classification of Diseases, 10th revision
ICU: Intensive care unit
IQR: Interquartile range
MAKE30: Major adverse events within 30 days
MAP: Mean arterial pressure
OPTIFLUID: OPTImized Restrictive Strategy Targeting Non-Resuscitative FLUIDs in Septic Shock
OR: Odds ratio
ROC: Receiver operating characteristics
RRT: Renal replacement therapy

## References

[1] Rhodes A, Evans LE, Alhazzani W, Levy MM, Antonelli M, Ferrer R (2017). Surviving sepsis campaign: international guidelines for management of sepsis and septic shock: 2016. Intensiv Care Med.

[2] Van Regenmortel N, Verbrugghe W, Roelant E, Van den Wyngaert T, Jorens PG (2018). Maintenance fluid therapy and fluid creep impose more significant fluid, sodium, and chloride burdens than resuscitation fluids in critically ill patients: a retrospective study in a tertiary mixed ICU population. Intensiv Care Med.

[3] Magee CA, Bastin MLT, Laine ME, Bissell BD, Howington GT, Moran PR (2018). Insidious harm of medication diluents as a contributor to cumulative volume and hyperchloremia: a prospective, open-label, sequential period pilot study. Crit Care Med.

[4] Finfer S, Bellomo R, Boyce N, French J, Myburgh J, Norton R (2004). A comparison of albumin and saline for fluid resuscitation in the intensive care unit. N Engl J Med.

[5] Myburgh JA, Finfer S, Bellomo R, Billot L, Cass A, Gattas D (2012). Hydroxyethyl starch or saline for fluid resuscitation in intensive care. N Engl J Med.

[6] Perner A, Haase N, Guttormsen AB, Tenhunen J, Klemenzson G, Aneman A (2012). Hydroxyethyl starch 130/042 versus ringer’s acetate in severe sepsis. N Engl J Med.

[7] Caironi P, Tognoni G, Masson S, Fumagalli R, Pesenti A, Romero M (2014). Albumin replacement in patients with severe sepsis or septic shock. N Engl J Med.

[8] Cruces P, Salas C, Lillo P, Salomon T, Lillo F, Hurtado DE (2014). The renal compartment: a hydraulic view. Intensiv Care Med Exp.

[9] Herrler T, Tischer A, Meyer A, Feiler S, Guba M, Nowak S (2010). The intrinsic renal compartment syndrome: new perspectives in kidney transplantation. Transplantation.

[10] Chowdhury AH, Cox EF, Francis ST, Lobo DN (2012). A randomized, controlled, double-blind crossover study on the effects of 2L infusions of 0.9% saline and plasma-lyte(R) 148 on renal blood flow velocity and renal cortical tissue perfusion in healthy volunteers. Ann Surg.

[11] Payen D, de Pont AC, Sakr Y, Spies C, Reinhart K, Vincent JL (2008). A positive fluid balance is associated with a worse outcome in patients with acute renal failure. Crit Care.

[12] Garzotto F, Ostermann M, Martin-Langerwerf D, Sanchez-Sanchez M, Teng J, Robert R (2016). The dose response multicentre investigation on fluid assessment (DoReMIFA) in critically ill patients. Crit Care.

[13] Zhang J, Crichton S, Dixon A, Seylanova N, Peng ZY, Ostermann M (2019). Cumulative fluid accumulation is associated with the development of acute kidney injury and non-recovery of renal function: a retrospective analysis. Crit Care Lond Engl.

[14] Hjortrup PB, Haase N, Bundgaard H, Thomsen SL, Winding R, Pettila V (2016). Restricting volumes of resuscitation fluid in adults with septic shock after initial management: the CLASSIC randomised, parallel-group, multicentre feasibility trial. Intensiv Care Med.

[15] Semler MW, Self WH, Wanderer JP, Ehrenfeld JM, Wang L, Byrne DW (2018). Balanced crystalloids versus saline in critically Ill adults. N Engl J Med.

[16] Self WH, Semler MW, Wanderer JP, Wang L, Byrne DW, Collins SP (2018). Balanced crystalloids versus saline in noncritically Ill adults. N Engl J Med.

[17] Asfar P, Meziani F, Hamel JF, Grelon F, Megarbane B, Anguel N (2014). High versus low blood-pressure target in patients with septic shock. N Engl J Med.

[18] Lamontagne F, Richards-Belle A, Thomas K, Harrison DA, Sadique MZ, Grieve RD (2020). Effect of reduced exposure to vasopressors on 90-day mortality in older critically Ill patients with vasodilatory hypotension: a randomized clinical trial. JAMA.

[19] Kellum JA, Lameire N, for the KDIGO AKI Guideline Work Group (2013). Diagnosis, evaluation, and management of acute kidney injury: a KDIGO summary (Part 1). Crit Care.

[20] Semler MW, Rice TW, Shaw AD, Siew ED, Self WH, Kumar AB (2016). Identification of major adverse kidney events within the electronic health record. J Med Syst.

[21] Zavada J, Hoste E, Cartin-Ceba R, Calzavacca P, Gajic O, Clermont G (2010). A comparison of three methods to estimate baseline creatinine for RIFLE classification. Nephrol Dial Transplant Off Publ Eur Dial Transpl Assoc Eur Ren Assoc.

[22] Therneau T, Atkinson B, Ripley B (2017). Rpart: recursive partitioning and regression trees. R Packag Ver.

[23] Boyd JH, Forbes J, Nakada TA, Walley KR, Russell JA (2011). Fluid resuscitation in septic shock: a positive fluid balance and elevated central venous pressure are associated with increased mortality. Crit Care Med.

[24] Sadaka F, Juarez M, Naydenov S, O’Brien J (2014). Fluid resuscitation in septic shock: the effect of increasing fluid balance on mortality. J Intensiv Care Med.

[25] Acheampong A, Vincent JL (2015). A positive fluid balance is an independent prognostic factor in patients with sepsis. Crit Care.

[26] Cronhjort M, Hjortrup PB, Holst LB, Joelsson-Alm E, Mårtensson J, Svensen C (2016). Association between fluid balance and mortality in patients with septic shock: a post hoc analysis of the TRISS trial. Acta Anaesthesiol Scand.

[27] de Oliveira FSV, Freitas FGR, Ferreira EM, de Castro I, Bafi AT, de Azevedo LCP (2015). Positive fluid balance as a prognostic factor for mortality and acute kidney injury in severe sepsis and septic shock. J Crit Care.

[28] Wong BT, Chan MJ, Glassford NJ, Martensson J, Bion V, Chai SY (2015). Mean arterial pressure and mean perfusion pressure deficit in septic acute kidney injury. J Crit Care.

[29] Palevsky PM, Molitoris BA, Okusa MD, Levin A, Waikar SS, Wald R (2012). Design of clinical trials in acute kidney injury: report from an NIDDK workshop on trial methodology. Clin J Am Soc Nephrol CJASN.

[30] Lindén-Søndersø A, Jungner M, Spångfors M, Jan M, Oscarson A, Choi S (2019). Survey of non-resuscitation fluids administered during septic shock: a multicenter prospective observational study. Ann Intensiv Care.

[31] Cecconi M, Hofer C, Teboul JL, Pettila V, Wilkman E, Molnar Z (2015). Fluid challenges in intensive care: the FENICE study: a global inception cohort study. Intensiv Care Med.

[32] Vaara ST, Ostermann M, Bitker L, Schneider A, Poli E, Hoste E (2021). Restrictive fluid management versus usual care in acute kidney injury (REVERSE-AKI): a pilot randomized controlled feasibility trial. Intensiv Care Med.

[33] Cinotti R, Lascarrou J-B, Azais M-A, Colin G, Quenot J-P, Mahé P-J (2021). Diuretics decrease fluid balance in patients on invasive mechanical ventilation: the randomized-controlled single blind. IRIHS study Crit Care.

[34] Mårtensson J, Bellomo R (2017). Does fluid management affect the occurrence of acute kidney injury?. Curr Opin Anaesthesiol.

[35] Skytte Larsson J, Bragadottir G, Krumbholz V, Redfors B, Sellgren J, Ricksten SE (2015). Effects of acute plasma volume expansion on renal perfusion, filtration, and oxygenation after cardiac surgery: a randomized study on crystalloid vs colloid. Br J Anaesth.

[36] Lengquist M, Lundberg OHM, Spångfors M, Annborn M, Levin H, Friberg H (2020). Sepsis is underreported in Swedish intensive care units: a retrospective observational multicentre study. Acta Anaesthesiol Scand.

