# Supplementary material for: Fluid accumulation and major adverse kidney events in sepsis: a multicenter observational study
Source: Ann Intensive Care. 2022 Jul 4;12:62. doi: 10.1186/s13613-022-01040-6 (PMC9250912; doi:10.1186/s13613-022-01040-6)
Supplement: Supplementary file 1 — Additional file 1: Detailed list of exposure variables; Filtering and processing of mean arterial pressure data; Description of Variable Importance analysis. [file 13613_2022_1040_MOESM1_ESM.pdf]

Additional Methods

Content

Detailed list of exposure variables………………………………………………………………..2
Filtering and processing of mean arterial pressure data………………………………..4
Description of Variable Importance analysis…………………………………………………5

Exposure variables collected at baseline or during exposure period.

Variable	Unit	Definition	Variable type
**Primary exposure variable**			
Cumulative fluid balance	ml or L	Cumulative fluid input minus cumulative fluid output (primary exposure variable) during the first two days in ICU	Continuous
**Baseline exposure variables**			
Age	years	Age on ICU admission	Continuous
Sex		Patient sex	Dichotomous
Body weight	kg	First recorded body weight in ICU	Continuous
Baseline creatinine	µmol/l	Determined by the following hierarchical approach:<br>1. First plasma creatinine value recorded in the ICU<br>2. Most recent creatinine level obtained within one year before ICU admission<br>3. Estimated by a three-variable equation	Continuous
Chronic liver disease		ICD-10 codes: K70.X, K72.1, K73.X, K74.X, K75.4, K76.0, K76.1, K76.6	Dichotomous
Chronic cardiac disease		ICD-10 codes: I25.X, I50.X	Dichotomous
Chronic respiratory disease		ICD-10 codes: J42.X, J43.X, J44.X, J45.X, J95.X	Dichotomous
Immune deficiency		ICD-10 codes: CXX.X, D83.0, D84.8, D84.9, T45.1, V43.3, Z51.1, Z85.X	Dichotomous
Location before ICU admission		Location before admission to ICU at hospital A-C for sepsis	Nominal:<br>1. Other ICU<br>2. Emergency Department<br>3. Recovery<br>4. Operating Room<br>5. High Dependency Unit<br>6. Ward
Admission hospital		First hospital in which the patient was admitted to ICU for sepsis	Nominal:<br>1. Hospital A<br>2. Hospital B<br>3. Hospital C
Admission year		2005-2018	Continuous
**Exposure variables reflecting illness severity during exposure period**			
Highest noradrenaline infusion rate	µg/kg/min	During the first two days in ICU	Ordinal:<br>1. ≤0.10<br>2. 0.11-0.20<br>3. >0.20
Vasopressin infusion			Dichotomous
Inotropic support		Continuous infusion of either adrenaline, dobutamine, levosimendan or milrinone	Dichotomous

Duration of MAP <65 mmHg	hours	The cumulative duration during the exposure period was calculated after filtering and processing of high-resolution data (see Table S2)	Ordinal:<br>1. <5<br>2. 5-10<br>3. >10
Invasive mechanical ventilation		Mechanical ventilation via endotracheal tube or tracheostomy	Dichotomous
Highest blood lactate level ≥2 mmol/l	mmol/l	Any level equal to or above 2 mmol/l. Measured on arterial blood on blood gas analyzer	Dichotomous
Highest bilirubin level	µmol/l	Measured at hospital laboratory	Ordinal:<br>1. <20<br>2. 20-32<br>3. >32
Lowest platelet count	$x10^9$	Measured at hospital laboratory	Ordinal:<br>1. ≥150<br>2. 100-149<br>3. <100
**Exposure to potentially nephrotoxic drugs during exposure period**			
Vancomycin		Any dose administered by intravenous injection or continuous infusion	Dichotomous
Aminoglycoside		Any intravenous dose of gentamicin, tobramycin, amikacin, or netilmicin	Dichotomous
Hydroxyethyl starch		Any volume of Hydroxyethyl Starch 130/0.4	Dichotomous
**Level of systemic inflammation during exposure period**			
Highest C-reactive protein level	mg/l	Measured at hospital laboratory	Continuous
**Other exposure variables potentially associated with both the primary exposure and outcome**			
Cumulative chloride dose	mmol	Calculated from all intravenous fluids	Ordinal:<br>1. <500<br>2. 500-999<br>3. ≥1000
Furosemide		Any dose administered by intravenous injection or continuous infusion	Dichotomous
Red blood cell transfusion		Administration of at least one unit	Dichotomous
Highest blood chloride level	mmol/l	Measured on arterial blood on blood gas analyzer and categorized based on data from previous publications[1-3]	Ordinal:<br>1. <108<br>2. 108-111<br>3. >111

Abbreviations: ICD-10, International Classification of Diseases, 10th revision

References:
1. Zhang Z, Xu X, Fan H, Li D, Deng H (2013) Higher serum chloride concentrations are associated with acute kidney injury in unselected critically ill patients. BMC Nephrol 28;14:235
2. Neyra JA, Canepa-Escaro F, Li X, Manllo J, Adams-Huet B, Yee J, Yessayan L (2015) Association of Hyperchloremia With Hospital Mortality in Critically Ill Septic Patients. Crit Care Med 43(9):1938-44
3. Suetrong B, Pisitsak C, Boyd JH, Russell JA, Walley KR (2016) Hyperchloremia and moderate increase in serum chloride are associated with acute kidney injury in severe sepsis and septic shock patients. Crit Care 6;20(1):315

Filtering and processing of mean arterial pressure data

Data Collection

The mean arterial pressure (MAP) was measured via an indwelling arterial line.
MAP measurements were automatically transferred to the Centricity Critical Care electronic patient data management system in intervals of 2 minutes on average.

Pre-filtering

1. Data points with MAP below 20 mmHg or above 250 mmHg were considered outliers due to artifacts and removed.
2. Patients with less than 50 recordings (between 20 and 250 mmHg) were not considered.
3. The time stamp of the first recording of each patient was set to be time 0 (zero) min.

Filtering

For each recoding *r* of each patient:

1. A window *w* of 20 minutes around the recording (10 minutes before and 10 minutes after) was considered. If there were less than 10 minutes before or after the recording, the maximum amount of available minutes were considered.
2. The median MAP *m* of the recordings in the window *w* was calculated.
3. The median absolute deviation (MAD) of the recordings in the window *w* was calculated. The MAD is the median absolute distance between the recordings in the window *w* and the median MAP *m*.
4. If the absolute distance between the recording *r* and the median MAP *m* was 4 times bigger than the MAD, the recording *r* was considered an outlier and removed.

Processing

After filtering, the cumulative time that a patient’s MAP was below 65 mmHg was calculated.

- If there were no recordings between two recordings with less than 65 mmHg, it was assumed the patient remained with a MAP below 65 mmHg for the time missing.
- The MAP between recordings below and above 65 mmHg was linearly interpolated to smooth the cumulative time.

Description of Variable Importance analysis

In a classification analysis such using the recursive partitioning function implemented in the 'rpart' R package (Therneau et al. 2017), the algorithm tries to find a rule c(x) consisting of binary splits that sorts the input population into different groups/classes.

In order to find the best splits, a loss function is minimized. The loss function measures how many observations have been classified correctly. Note that a variable x_i, i=1,…,n can be split several times. An overall measure of variable importance is the sum of the goodness of split measures (obtained with the loss function) for each split for which it was the primary variable, plus goodness * (adjusted agreement) for all splits in which it was a surrogate (=help to account for missing values). In order to calculate the relative importance, the importance of each variable was divided by the total importance obtained by summing over all variables considered.

The loss function is also called 0-1 loss function in this context since it is based on a sum of zeros and ones: 1 if the observation was correctly classified and 0 if not. If the sum is divided by the number of observation it is also referred to as error rate.

Reference:
Therneau, T, Atkinson B, Ripley B (2017) rpart: Recursive Partitioning and Regression Trees.,. https://CRAN.R-project.org/package=rpart, r package version 4.1-11.
