# Supplementary material for: Fluid accumulation and major adverse kidney events in sepsis: a multicenter observational study
Source: Ann Intensive Care. 2022 Jul 4;12:62. doi: 10.1186/s13613-022-01040-6 (PMC9250912; doi:10.1186/s13613-022-01040-6)
Supplement: Supplementary file 2 — Additional file 2: Figure S1. Cumulative fluid balance by admission year, Figure S2. Restricted cubic spline curves, Table S1. Backwards selection logistic regression adjusted for early AKI, Table S2. Backwards selection logistic regression adjusted for fluid input and urine output, Table S3. Forward selection logistic regression (association with MAKE30), Table S4. Backwards selection logistic regression (association with RRT or sustained renal dysfunction), Table S5. Forward selection logistic regression (association with RRT or sustained renal dysfunction). [file 13613_2022_1040_MOESM2_ESM.pdf]

Additional Results

Content

Figure S1. Cumulative fluid balance by admission year……………………………………..2
Figure S2. Restricted cubic spline curves…………………………………………………………3
Table S1. Backwards selection logistic regression adjusted for early AKI……………4
Table S2. Backwards selection logistic regression adjusted for fluid input and urine output…………………………………………………………………………………………………5
Table S3. Forward selection logistic regression (association with MAKE30)……….6
Table S4. Backwards selection logistic regression (association with RRT or sustained renal dysfunction)………………………………………………………………………….7
Table S5. Forward selection logistic regression (association with RRT or sustained renal dysfunction) ……………………………………………………………………….8

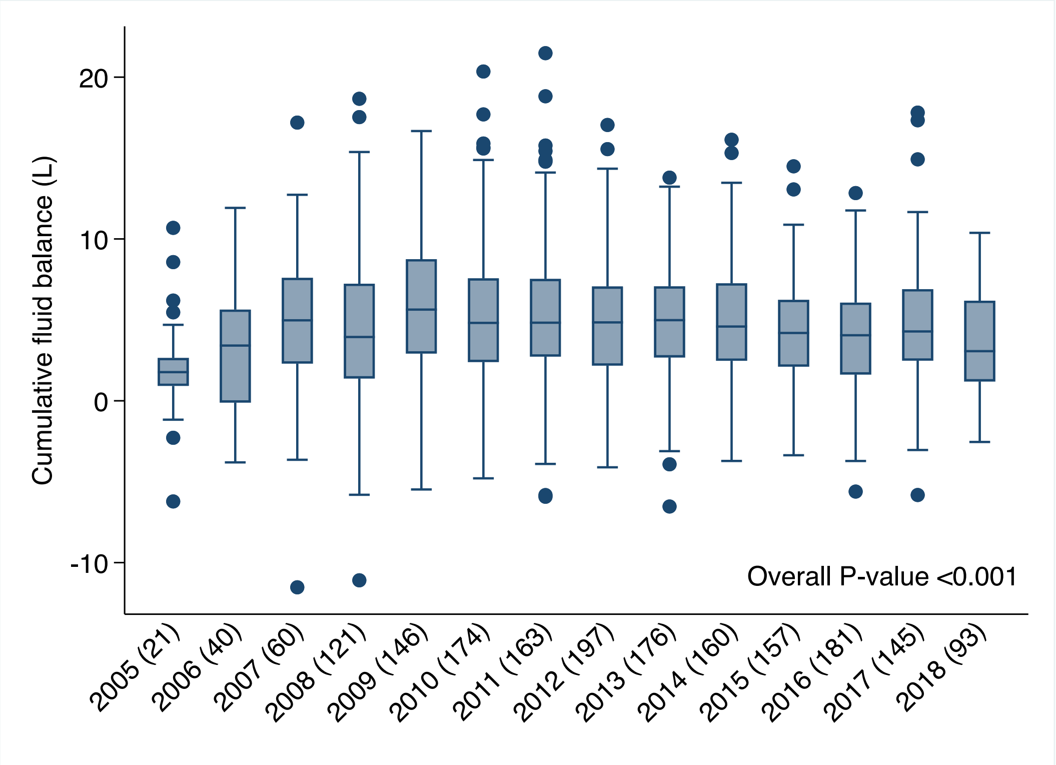

Figure S1. Box plot showing the cumulative fluid balance by admission year. Number of patients is indicated in parenthesis.

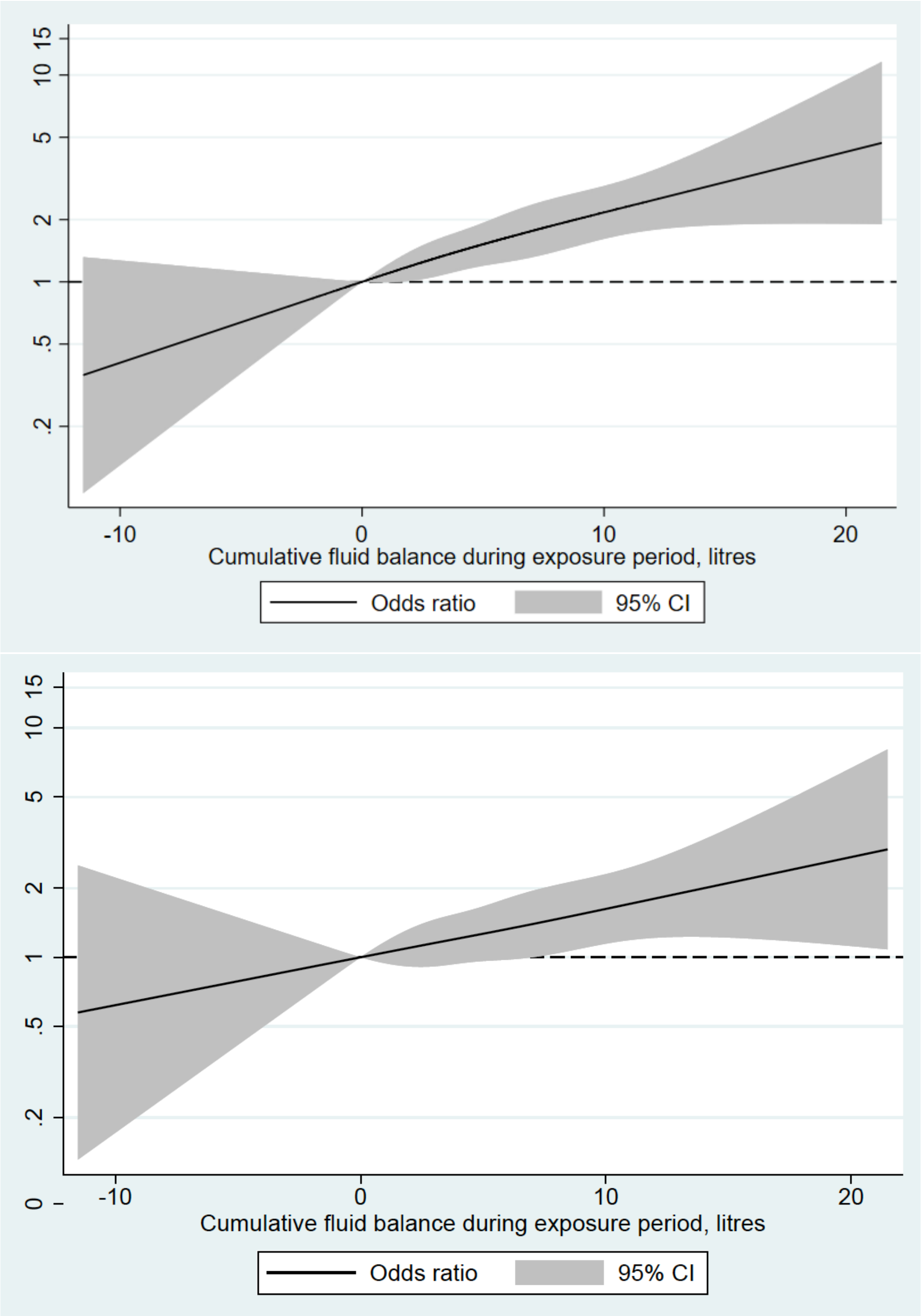

Figure S2. Unadjusted (upper panel) and adjusted (lower panel) restricted cubic spline curve showing the odds ratio and 95% confidence intervals for MAKE30 risk by cumulative fluid balance. All odds ratios are with respect to a reference fluid balance of zero litres.

Table S1. Multivariable logistic regression analysis showing the association with major adverse kidney events within 30 days.

Variable	Unadjusted Odds Ratio (95% CI)	P value	Adjusted Odds Ratio[a] (95% CI)	P value
Cumulative fluid balance, litre	1.08 (1.05-1.11)	<0.001	1.04 (1.003-1.07)	0.03
Age, year	1.02 (1.01-1.03)	<0.001	1.03 (1.02-1.04)	<0.001
Male sex	0.92 (0.75-1.13)	0.41	0.86 (0.67-1.11)	0.26
Body weight, kg	0.99 (0.99-1.00)	0.01	0.99 (0.99-1.00)	0.04
Acute kidney injury[b]	2.32 (1.85-2.90)	<0.001	2.03 (1.56-2.63)	<0.001
Chronic liver disease	2.21 (1.58-3.10)	<0.001	1.78 (1.17-2.72)	0.007
Chronic cardiac disease	1.37 (1.06-1.78)	0.02	1.32 (0.95-1.83)	0.10
Chronic respiratory disease	1.26 (0.93-1.73)	0.14	1.19 (0.82-1.73)	0.37
Immune deficiency	1.71 (1.39-2.12)	<0.001	1.58 (1.22-2.05)	0.001
Duration with MAP <65 mmHg				
<5 h	1.00		1.00	
5-10 h	0.90 (0.68-1.18)	0.44	0.75 (0.55-1.03)	0.08
>10 h	1.16 (0.92-1.46)	0.21	0.87 (0.66-1.14)	0.32
Vancomycin therapy	1.51 (1.04-2.18)	0.03	1.77 (1.15-2.73)	0.01
Aminoglycoside therapy	1.00 (0.78-1.31)	0.95	0.86 (0.63-1.17)	0.33
Hydroxyethyl starch administration	0.95 (0.72-1.26)	0.74	0.92 (0.63-1.33)	0.65
Highest C-reactive protein, mg/l	1.00 (1.00-1.00)	0.23	1.00 (1.00-1.00)	0.50
Highest chloride level				
<108 mmol/l	1.00		1.00	
108-111 mmol/l	0.88 (0.68-1.15)	0.36	0.90 (0.66-1.24)	0.53
>111 mmol/l	0.82 (0.64-1.06)	0.13	0.79 (0.58-1.08)	0.14
Highest lactate level				
<2 mmol/l	1.00		1.00	
≥2 mmol/l	1.46 (1.13-1.89)	0.004	1.03 (0.74-1.42)	0.88
Highest bilirubin level				
<20 µmol/l	1.00		1.00	
20-32 µmol/l	1.24 (0.95-1.62)	0.11	1.06 (0.78-1.43)	0.73
>32 µmol/l	1.79 (1.40-2.29)	<0.001	1.27 (0.95-1.71)	0.11
Lowest platelet count				
≥150	1.00		1.00	
100-149	1.50 (1.14-1.99)	0.004	1.66 (1.20-2.29)	0.002
<100	2.21 (1.75-2.80)	<0.001	2.08 (1.55-2.79)	<0.001
Invasive mechanical ventilation	1.37 (1.12-1.69)	0.003	1.62 (1.26-2.10)	<0.001

[a]The model included 1641 patients with complete data and was also adjusted for admission source, admission hospital, and admission year. Model Area under the receiver operating characteristics curve 0.74
[b]Defined based on changes in plasma creatinine and/or oliguria according to the Kidney Disease: Improving Global Outcomes (KDIGO) criteria

Table S2. Multivariable logistic regression analysis showing the association with major adverse kidney events within 30 days.

Variable	Unadjusted Odds Ratio (95% CI)	P value	Adjusted Odds Ratio[a] (95% CI)	P value
Total fluid input, litre	1.00 (0.97-1.03)	0.96	0.98 (0.94-1.02)	0.23
Total urine output, litre	0.82 (0.78-0.86)	<0.001	0.84 (0.79-0.89)	<0.001
Age, year	1.02 (1.01-1.03)	<0.001	1.03 (1.02-1.04)	<0.001
Male sex	0.92 (0.75-1.13)	0.41	0.88 (0.68-1.14)	0.33
Body weight, kg	0.99 (0.99-1.00)	0.01	1.00 (0.99-1.00)	0.28
Baseline creatinine, per 10 µmol/l	1.00 (1.00-1.01)	0.02	1.01 (1.00-1.02)	0.16
Chronic liver disease	2.21 (1.58-3.10)	<0.001	1.63 (1.08-2.47)	0.02
Chronic cardiac disease	1.37 (1.06-1.78)	0.02	1.24 (0.89-1.72)	0.21
Chronic respiratory disease	1.26 (0.93-1.73)	0.14	1.17 (0.80-1.71)	0.43
Immune deficiency	1.71 (1.39-2.12)	<0.001	1.65 (1.27-2.14)	<0.001
Duration with MAP <65 mmHg				
<5 h	1.00		1.00	
5-10 h	0.90 (0.68-1.18)	0.44	0.74 (0.54-1.01)	0.06
>10 h	1.16 (0.92-1.46)	0.21	0.90 (0.69-1.19)	0.47
Vancomycin therapy	1.51 (1.04-2.18)	0.03	1.83 (1.18-2.84)	0.007
Aminoglycoside therapy	1.00 (0.78-1.31)	0.95	0.94 (0.69-1.29)	0.70
Hydroxyethyl starch administration	0.95 (0.72-1.26)	0.74	0.94 (0.65-1.38)	0.77
Highest C-reactive protein, mg/l	1.00 (1.00-1.00)	0.23	1.00 (1.00-1.00)	0.56
Highest chloride level				
<108 mmol/l	1.00		1.00	
108-111 mmol/l	0.88 (0.68-1.15)	0.36	0.91 (0.66-1.26)	0.57
>111 mmol/l	0.82 (0.64-1.06)	0.13	0.80 (0.58-1.09)	0.16
Highest lactate level				
<2 mmol/l	1.00		1.00	
≥2 mmol/l	1.46 (1.13-1.89)	0.004	1.12 (0.81-1.55)	0.49
Highest bilirubin level				
<20 µmol/l	1.00		1.00	
20-32 µmol/l	1.24 (0.95-1.62)	0.11	1.07 (0.79-1.46)	0.65
>32 µmol/l	1.79 (1.40-2.29)	<0.001	1.39 (1.03-1.88)	0.03
Lowest platelet count				
≥150	1.00		1.00	
100-149	1.50 (1.14-1.99)	0.004	1.76 (1.27-2.43)	0.001
<100	2.21 (1.75-2.80)	<0.001	2.29 (1.70-3.07)	<0.001
Invasive mechanical ventilation	1.37 (1.12-1.69)	0.003	1.65 (1.27-2.13)	<0.001

[a]The model included 1641 patients with complete data and was also adjusted for admission source, admission hospital, and admission year. Model Area under the receiver operating characteristics curve 0.74

Table S3. Univariable (Model I) and multivariable (Model II-VII) forward selection logistic regression analyses showing the association between cumulative fluid balance (in litres) and major adverse kidney events within 30 days

Model	N	df	OR (95% CI)	AIC
I	1641	2	1.08 (1.05-1.11)	1934
II	1641	7	1.07 (1.04-1.11)	1895
III	1641	11	1.08 (1.05-1.11)	1853
IV	1641	17	1.06 (1.03-1.09)	1833
V	1641	24	1.07 (1.03-1.10)	1835
VI	1641	33	1.05 (1.02-1.09)	1808
VII	1641	38	1.05 (1.02-1.09)	1816

Abbreviations: df, degree of freedom; OR, odds ratio; AIC, Akaike's information criterion

Model I was unadjusted

Model II was adjusted for age, sex, body weight, baseline creatinine and admission year

Model III was adjusted for Model II variables, and comorbidities

Model IV was adjusted for Model III variables, lactate >2 mmol/l, platelet categories, bilirubin categories and highest CRP

Model V was adjusted for Model IV variables, MAP duration categories, chloride level categories, and nephrotoxic drugs

Model VI was adjusted for Model V variables, admission hospital, admission source, mechanical ventilation, and red blood cell transfusion

Model VII was adjusted for Model VI variables, vasopressor categories, inotropic support, and furosemide administration

Table S4. Multivariable logistic regression analysis showing the association with the composite outcome of renal replacement therapy or sustained renal dysfunction (a last inpatient plasma creatinine level ≥200% of baseline) within 30 days

**Variable**	**Unadjusted Odds Ratio (95% CI)**	**P value**	**Adjusted Odds Ratio[a] (95% CI)**	**P value**
Cumulative fluid balance, litre	1.07 (1.03-1.11)	0.001	1.06 (1.01-1.11)	0.01
Age, year	0.98 (0.97-0.99)	<0.001	0.99 (0.98-1.00)	0.02
Male sex	1.24 (0.90-1.72)	0.19	1.09 (0.74-1.60)	0.65
Body weight, kg	1.01 (1.00-1.02)	0.01	1.01 (1.00-1.02)	0.04
Baseline creatinine, per 10 µmol/l	1.00 (0.99-1.02)	0.60	1.01 (0.99-1.02)	0.22
Chronic liver disease	2.13 (1.34-3.39)	0.001	1.33 (0.79-2.25)	0.28
Chronic cardiac disease	0.78 (0.49-1.23)	0.28	0.83 (0.48-1.41)	0.49
Chronic respiratory disease	0.96 (0.57-1.60)	0.86	1.23 (0.68-2.22)	0.49
Immune deficiency	1.40 (1.01-1.94)	0.04	1.45 (0.98-2.16)	0.06
Duration with MAP <65 mmHg				
<5 h	1.00		1.00	
5-10 h	1.15 (0.77-1.72)	0.49	1.07 (0.70-1.63)	0.77
>10 h	0.92 (0.63-1.33)	0.64	0.76 (0.50-1.15)	0.19
Vancomycin therapy	1.85 (1.12-3.07)	0.02	1.56 (0.86-2.83)	0.14
Aminoglycoside therapy	1.23 (0.83-1.81)	0.30	1.38 (0.91-2.10)	0.13
Hydroxyethyl starch administration	0.98 (0.63-1.51)	0.91	1.01 (0.58-1.74)	0.98
Highest C-reactive protein, mg/l	1.00 (1.00-1.00)	0.50	1.00 (1.00-1.00)	0.30
Highest chloride level				
<108 mmol/l	1.00		1.00	
108-111 mmol/l	1.01 (0.68-1.52)	0.95	0.86 (0.55-1.34)	0.49
>111 mmol/l	0.79 (0.53-1.19)	0.26	0.65 (0.41-1.02)	0.06
Highest lactate level				
<2 mmol/l	1.00		1.00	
≥2 mmol/l	1.26 (0.84-1.89)	0.27	1.05 (0.65-1.70)	0.85
Highest bilirubin level				
<20 µmol/l	1.00		1.00	
20-32 µmol/l	1.68 (1.11-2.55)	0.02	1.46 (0.94-2.28)	0.10
>32 µmol/l	2.04 (1.39-3.01)	<0.001	1.29 (0.84-1.98)	0.25
Lowest platelet count				
≥150	1.00		1.00	
100-149	1.31 (0.83-2.08)	0.25	1.18 (0.71-1.96)	0.52
<100	1.93 (1.33-2.80)	0.001	1.54 (1.00-2.37)	0.05
Invasive mechanical ventilation	1.71 (1.23-2.38)	0.002	1.85 (1.28-2.67)	0.001

[a]The model included 1641 patients with complete data and was also adjusted for admission source, admission hospital, and admission year. Model Area under the receiver operating characteristics curve 0.71

Table S5. Univariable (Model I) and multivariable (Model II-VII) forward selection logistic regression analyses showing the association between cumulative fluid balance (in litres) and the composite outcome of renal replacement therapy or sustained renal dysfunction (a last inpatient plasma creatinine level ≥200% of baseline) within 30 days

Model	N	df	OR (95% CI)	AIC
I	1641	2	1.08 (1.04-1.13)	989
II	1641	7	1.09 (1.04-1.14)	982
III	1641	11	1.09 (1.04-1.14)	978
IV	1641	17	1.07 (1.03-1.12)	983
V	1641	24	1.07 (1.03-1.12)	988
VI	1641	28	1.06 (1.01-1.11)	982
VII	1641	33	1.06 (1.01-1.12)	982

Abbreviations: df, degree of freedom; OR, odds ratio; AIC, Akaike's information criterion

Model I was unadjusted

Model II was adjusted for age, sex, body weight, baseline creatinine and admission year

Model III was adjusted for Model II variables, and comorbidities

Model IV was adjusted for Model III variables, lactate >2 mmol/l, platelet categories, bilirubin categories and highest CRP

Model V was adjusted for Model IV variables, MAP duration categories, chloride level categories, and nephrotoxic drugs

Model VI was adjusted for Model V variables, admission hospital, admission source, mechanical ventilation, and red blood cell transfusion

Model VII was adjusted for Model VI variables, vasopressor categories, inotropic support, and furosemide administration
